# Anaerobic Reduction of Nitrate to Nitrous Oxide Is Lower in *Bradyrhizobium japonicum* than in *Bradyrhizobium diazoefficiens*

**DOI:** 10.1264/jsme2.ME17081

**Published:** 2017-11-03

**Authors:** Arthur Fernandes Siqueira, Kiwamu Minamisawa, Cristina Sánchez

**Affiliations:** 1 Graduate School of Life Sciences, Tohoku University 2–2–1 Katahira, Aoba-ku, Sendai 980–8577 Japan

**Keywords:** denitrification, *Bradyrhizobium*, nitrogen cycle, bacterial physiology

## Abstract

When soil oxygen levels decrease, some bradyrhizobia use denitrification as an alternative form of respiration. *Bradyrhizobium diazoefficiens* (*nos*^+^) completely denitrifies nitrate (NO_3_^−^) to dinitrogen, whereas *B. japonicum* (*nos*^−^) is unable to reduce nitrous oxide to dinitrogen. We found that anaerobic growth with NO_3_^−^ as the electron acceptor was significantly lower in *B. japonicum* than in *B. diazoefficiens*, and this was not explained by the absence of *nos* in *B. japonicum*. Our results indicate that the reason for the limited growth of *B. japonicum* is weak NO_3_^−^ reduction due to impaired periplasmic nitrate reductase activity, which may rely on posttranscriptional events.

Rhizobia are gram-negative α-proteobacteria with the ability to fix dinitrogen (N_2_) in symbiosis with leguminous plants (16, 28). Soybean generally accommodates rhizobia from the genera *Bradyrhizobium* (2). If oxygen levels drop, some bradyrhizobial species use denitrification as alternative respiration and sequentially reduce nitrate (NO_3_^−^) to nitrous oxide (N_2_O) or N_2_ (5, 11, 15, 19, 29). Bradyrhizobial denitrification starts with the reduction of NO_3_^−^ to nitrite (NO_2_^−^) by the periplasmic nitrate reductase (Nap) encoded by *napA* in the *napEDABC* gene cluster (5). The second step is the reduction of NO_2_^−^ to nitric oxide (NO) by the copper-containing nitrite reductase encoded by *nirK* (5). The reduction of NO to N_2_O occurs through a *c*-type NO reductase, the cytochrome *bc* complex NorCB encoded by *norECBQD* (5, 26). N_2_O is then reduced to N_2_ by the N_2_O reductase NosZ encoded in the *nosRZDFYLX* gene cluster (5).

*Bradyrhizobium diazoefficiens* (formerly classified into *B. japonicum*) (9) has a complete set of denitrification genes, whereas *B. japonicum* lacks the *nos* gene cluster and releases N_2_O as the final product of denitrification (12, 14, 19, 24). Therefore, *B. diazoefficiens* and *B. japonicum* share the first three steps of denitrification (reduction of NO_3_^−^ to N_2_O). Shiina *et al.* (23) demonstrated that the occurrence of bradyrhizobia with *nosZ* (*nosZ*^+^) or without *nosZ* (*nosZ*^−^) in Japanese soybean fields largely depended on the soil type. Andosol, a volcanic soil composed of porous sediments (3, 25), is significantly dominated by *nosZ*^−^, whereas Gleysol, a wetland soil saturated with groundwater, in which the water regime favors low-oxygen conditions (13), is significantly dominated by *nosZ*^+^ (23). These findings suggested that *B. diazoefficiens* (*nosZ*^+^) is predominant in Gleysol soils and *B. japonicum* (*nosZ*^−^) in Andosols (23). Saeki *et al.* (18) recently indicated that the possession of *nosZ* confers a competitive advantage to *B. diazoefficiens* in flooded soil; this is consistent with the predominance of *nosZ*^+^ bradyrhizobia in Gleysol soils (23). Thus, the aim of the present study was to identify key physiological traits in denitrification that differentiate the distribution of *B. diazoefficiens* and *B. japonicum* in soybean fields in Japan.

The *Bradyrhizobium* strains used in this study are listed in Table S1. Cells were precultured at 30°C for 72 h in HM salt medium (8) supplemented with 0.1% l-(+)-arabinose and 0.25% (w/v) yeast extract. HM medium supplemented with trace metals (20) and 10 mM KNO_3_ (HMMN medium) was employed in denitrification assays. In growth experiments, precultured cells were inoculated into 34-mL test tubes containing 5 mL HMMN medium. Initial optical density at 660 nm was adjusted to 0.01. Foam stoppers were used for the aerobic treatment and butyl rubber stoppers for the anaerobic and microaerobic treatments. In the anaerobic treatment, the gas phase was replaced with 100% N_2_ in a vacuum line. In the microaerobic treatment, the gas phase (2% O_2_ [v/v], N_2_ balance) was replaced daily. Cells were grown at 30°C with reciprocal shaking at 300 rpm. Dissolved oxygen levels were verified in each treatment with a 5300A Biological Oxygen Monitor (Yellow Springs Instruments, Yellow Springs, OH, USA) (Fig. S1). Growth was monitored daily by measuring the optical density of the cultures at 660 nm; the number of cells was assessed by direct counting with a 20-μm-deep hemocytometer (Sunlead Glass, Saitama, Japan) and an Olympus BX51 Fluorescence Microscope (Olympus, Tokyo, Japan).

In denitrification assays, precultured cells were inoculated into 100-mL vials containing 20 mL of HMMN at an initial optical density (660 nm) of 0.02, and grown anaerobically at 30°C with reciprocal shaking at 100 rpm. Extracellular NO_3_^−^ concentrations were assessed using a Dionex ICS-1100 Basic Integrated Ion Chromatography System (Thermo Fisher Scientific, Waltham, MA, USA). Prior to injections, each sample was filtered through a Minisart syringe filter (pore size, 0.2 μm; Sartorius, Göttingen, Germany) and diluted with Milli-Q water. In N_2_O measurements, 0.2 mL of the gas phase was injected into a GC-17A Gas Chromatograph (Shimadzu, Kyoto, Japan) as described previously (19). Methyl viologen–dependent nitrate reductase activity was measured as described by Sánchez and co-workers (21).

The isolation of total RNA, the DNaseI treatment, and cDNA synthesis were performed as described previously (4, 10). Prior to cDNA synthesis, the absence of DNA in DNaseI-treated RNA was confirmed by PCR with the *sigA* primer pair (Table S2). Relative expression was analyzed by quantitative reverse-transcription PCR in a LightCycler Nano Instrument (Roche, Basel, Switzerland) using FastStart Essential DNA Green Master (Roche) and specific primers for *sigA*, *napA*, *nirK*, and *norB* (Table S2). The PCR program was set according to the manufacturer’s instructions, and the specificity of PCR amplification was confirmed by a melting-curve analysis. The relative expression of the target genes was calculated by the 2^−ΔΔCT^ method (22) using *sigA* as an internal control.

The anaerobic growth of *B. japonicum* USDA 6^T^ and CPAC 15 with NO_3_^−^ as the electron acceptor was significantly lower than that of *B. diazoefficiens* USDA 110^T^ and CPAC 7 based on the means of optical density and cell number (Fig. 1A; Fig. S2). However, no significant differences were observed between the growth of *B. japonicum* and *B. diazoefficiens* strains under aerobic or microaerobic conditions in the presence of NO_3_^−^ (Fig. S2). Since *B. japonicum* lacks the *nos* gene cluster (14, 24), we investigated whether N_2_O reductase, encoded by *nosZ*, supports fast growth by *B. diazoefficiens* USDA 110^T^. The growth of the *nosZ* mutant USDA 110^T^ (USDA 110^T^-*nosZ*) was similar to that of wild-type USDA 110^T^, indicating that the N_2_O reduction step did not markedly contribute to the difference observed in growth between *B. japonicum* and *B. diazoefficiens* under anaerobic NO_3_^−^-respiring conditions (Fig. 1A). In terms of bioenergetics, there are few disadvantages for cells failing to perform the final step of N_2_O reduction (17). In contrast, Saeki *et al.* suggested that *nosZ* confers a competitive advantage in flooded soils (18). Soil factors may influence the relevance of the N_2_O reduction step in bradyrhizobial competition.

In order to examine whether the difference in growth extends to the species level, we randomly selected 11 strains of *B. japonicum* and 15 strains of *B. diazoefficiens* (Table S2). The phylogenetic tree of these strains is shown in Fig. S3. In the presence of NO_3_^−^, the mean growth of *B. japonicum* strains was significantly lower than that of *B. diazoefficiens* strains under anaerobiosis (Fig. 1B; Table S3), but not under aerobiosis or microaerobiosis (Fig. 1C and D; Table S3). Thus, low growth appeared to be a general phenomenon in *B. japonicum*.

In order to compare the steps of denitrification that were common to *B. japonicum* and *B. diazoefficiens* (*i.e.* reduction of NO_3_^−^ to N_2_O), we monitored NO_3_^−^ and N_2_O concentrations in batch cultures of *B. japonicum* USDA 6^T^ and CPAC 15 and *B. diazoefficiens* USDA 110^T^ and CPAC 7 under anaerobic NO_3_^−^-respiring conditions. The *B. diazoefficiens* USDA 110^T^-*nosZ* mutant was used as a control equivalent to *B. japonicum* in terms of the reduction of NO_3_^−^ to N_2_O. The three strains of *B. diazoefficiens* had completely consumed NO_3_^−^ by day 7 (Fig. 2A). On the other hand, N_2_O production was observed exclusively in the gas phase of the USDA 110^T^-*nosZ* mutant culture (Fig. 2B), which likely reflected the stoichiometric conversion of 2 moles of NO_3_^−^ to 1 mole of N_2_O (Fig. 2A and B). The two strains of *B. japonicum* consumed less NO_3_^−^ (Fig. 2C) and produced less N_2_O (Fig. 2D) than the USDA 110^T^-*nosZ* mutant. These results indicate that *B. japonicum* is less capable of reducing NO_3_^−^ to N_2_O than *B. diazoefficiens*.

Nap activity was markedly weaker in *B. japonicum* (USDA 6^T^ and CPAC 15) than in *B. diazoefficiens* (USDA 110^T^, CPAC 7, and USDA 110^T^-*nosZ*) under anaerobic NO_3_^−^-respiring conditions (Fig. 3A); *B. japonicum* USDA 6^T^ and CPAC 15 showed no activity at 48 h and low activity at 72 h (Fig. 3A). This may explain the low rates of NO_3_^−^ consumption and N_2_O production in *B. japonicum* (Fig. 2C and D). We found that NapA and NapB (the catalytic and electron-transfer subunits of Nap, respectively) amino acid sequences shared 94–97% identity among USDA 110^T^, USDA 6^T^, and CPAC 15 and conserved the motifs involved in catalysis (29), which suggest the functional conservation of Nap in *B. japonicum*. Thus, the lower Nap activity in *B. japonicum* may rely on differences during the expression process. We then assessed the *napA* transcript level in each of the strains relative to that in USDA 110^T^. Although transcript levels varied among strains, *napA* expression was not significantly different between *B. diazoefficiens* and *B. japonicum* (Fig. 3A). Collectively, these results suggest that the low efficiency for NO_3_^−^ reduction in *B. japonicum* relies on posttranscriptional events. Similarly, *Sinorhizobium meliloti* is unable to grow under anaerobic NO_3_^−^-respiratory conditions even though denitrification genes are fully induced (27). In addition, *Agrobacterium tumefaciens* and *Pseudomonas* sp. G59 are unable to make an effective switch to denitrification in the absence of oxygen (1, 6).

We also analyzed *nirK* and *norB* transcript levels relative to that of the *napA* transcript for each strain. The levels of both transcripts were higher in *B. japonicum* (Fig. 3B), even though *B. japonicum* reduced less NO_3_^−^ than *B. diazoefficiens* (Fig. 2C; Fig. 3A). A possible explanation is that *B. japonicum* overexpresses *nirK* and *norB* to compensate for the lower activity of Nap. This induction may be dependent on the FixLJ–FixK_2_–NnrR regulatory cascade (5, 7), which controls the expression of denitrification genes in *B. diazoefficiens*. *B. japonicum* USDA 6^T^ and CPAC 15 conserved a complete set of these regulatory genes and binding sites for FixK/FNR (fumarate and nitrate reductase regulator) regulators (5) in the promoter regions of *napE*, *nirK*, and *norC* genes (data not shown). However, we cannot exclude the possibility of different regulatory networks in *B. japonicum*.

Our results show that the low efficiency for NO_3_^−^ reduction, as a consequence of impaired Nap activity, is the main factor limiting the growth of *B. japonicum* under anaerobic NO_3_^−^-respiring conditions, which cannot be interpreted at the expression level of the genes responsible for denitrification. If confronted with oxygen depletion, the activation of NO_3_^−^ reduction may be crucial for preventing anoxia entrapment by providing energy for the biosynthesis of the entire denitrification proteome (6, 7, 27). *B. japonicum* may be less competitive than *B. diazoefficiens* due to energy depletion under anaerobic conditions, despite sufficient *nap* expression, which may contribute to the predominance of *B. diazoefficiens* in Gleysol (23).

## Supplemental material



## Figures and Tables

**Fig. 1:**
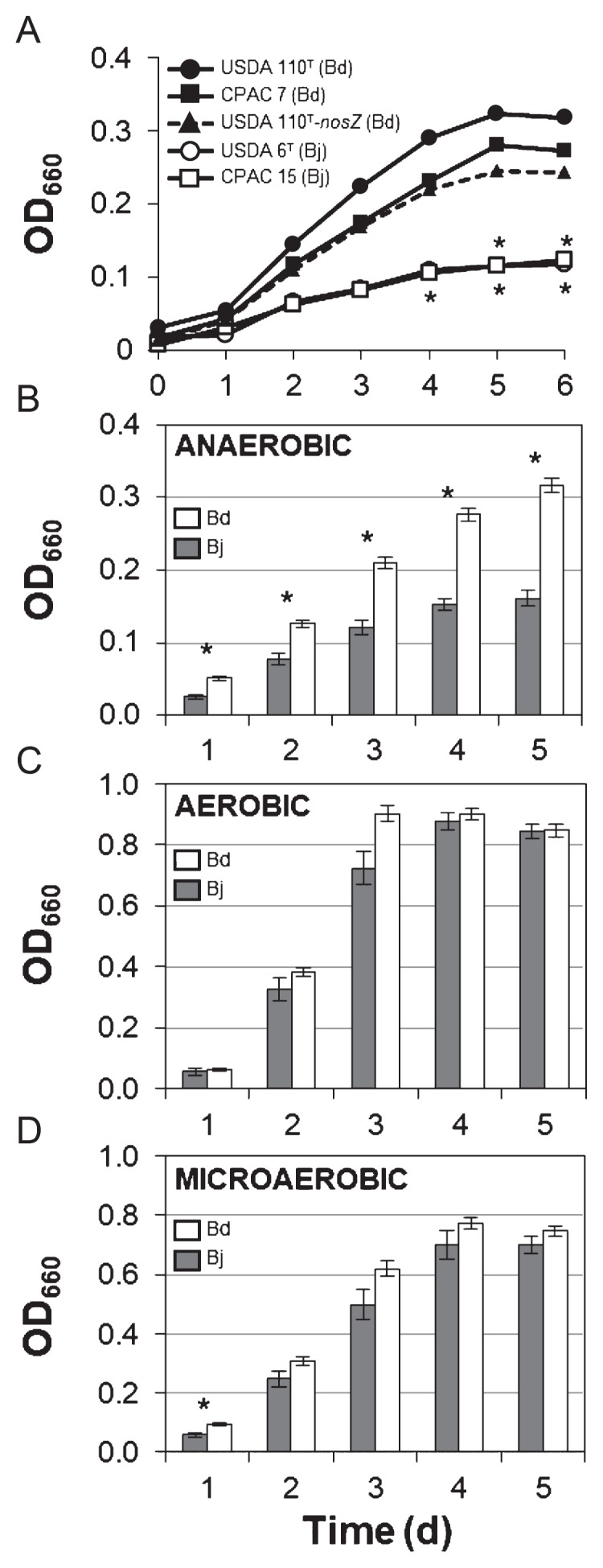
Growth of *Bradyrhizobium japonicum* and *B. diazoefficiens*. (A) Anaerobic growth of *B. japonicum* (USDA 6^T^ and CPAC 15) and *B. diazoefficiens* (USDA 110^T^, CPAC 7, and USDA 110^T^*-nosZ* mutant) in HMMN medium. *Values significantly different from those of *B. diazoefficiens* USDA 110^T^ (*t*-test, *P*<0.05; *n*=3). (B–D) Average growth profiles of *B. diazoefficiens* (15 strains) and *B. japonicum* (11 strains) at the indicated conditions in HMMN medium; error bars indicate SE. *Values significantly different between *B. diazoefficiens* and *B. japonicum* (*t*-test, *P*<0.001; *n*=3). Bd, *B. diazoefficiens*; Bj, *B. japonicum*.

**Fig. 2:**
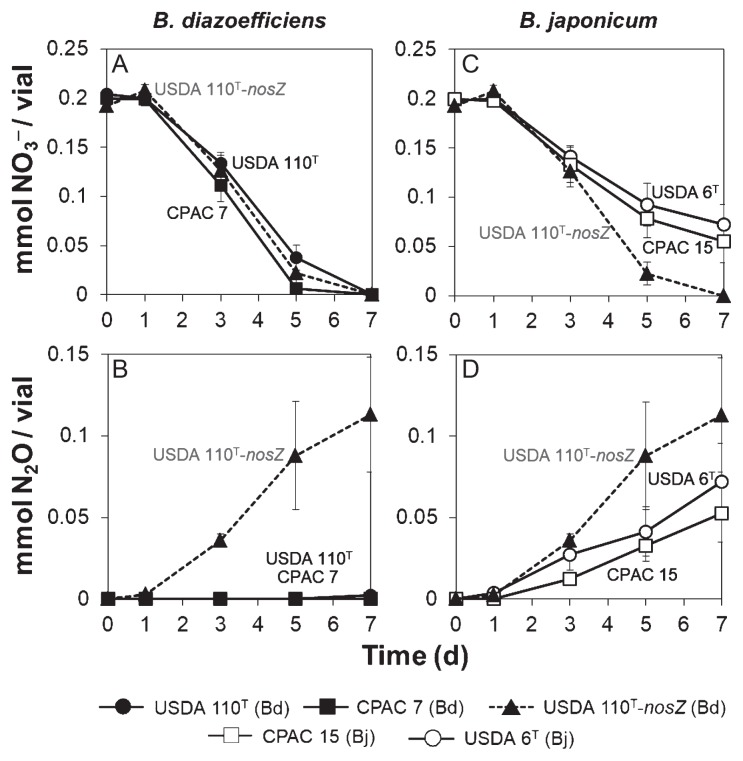
Nitrate (NO_3_^−^) consumption (A, C) and nitrous oxide (N_2_O) production (B, D) by *B. diazoefficiens* and *B. japonicum* strains growing under anaerobiosis in HMMN medium. *B. diazoefficiens* USDA 110^T^-*nosZ* is shown as a reference; data are the means of three different starter cultures; error bars indicate SE. Bd, *B. diazoefficiens*; Bj, *B. japonicum*.

**Fig. 3:**
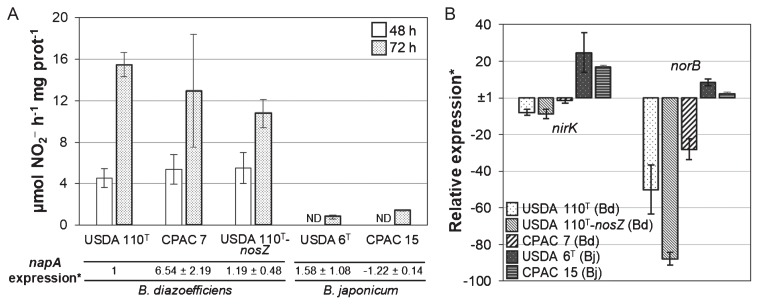
Periplasmic nitrate reductase activity and relative expression of *napA*, *nirK*, and *norB*. (A) Methyl viologen–dependent nitrate reductase activity (top) and relative expression of *napA* (bottom) in the indicated strains. Activities were measured after an incubation for 48 or 72 h under anaerobiosis in HMMN medium; data are the means±SE of three different starter cultures. The expression of *napA* was measured at 48 h; data are the means±SE of three independent RNA samples. *napA expression values are relative to that of USDA 110^T^, which was set at 1. ND, not detected. (B) Relative expression of *nirK* and *norB* in the indicated strains; data are the means±SE of three independent RNA samples; *Values are relative to the *napA* value of each strain. Bd, *B. diazoefficiens*; Bj, *B. japonicum*. In A and B, up-regulated (1>2^−ΔΔCT^) or down-regulated (1>2^−ΔΔCT^>0) expression is indicated as 1/2^−ΔΔCT^ or −1/2^−ΔΔCT^, respectively.
