# Supplementary material for: Anaerobic Reduction of Nitrate to Nitrous Oxide Is Lower in Bradyrhizobium japonicum than in Bradyrhizobium diazoefficiens
Source: Microbes Environ. 2017 Nov 3;32(4):398–401. doi: 10.1264/jsme2.ME17081 (PMC5745027; doi:10.1264/jsme2.ME17081)
Supplement: Supplementary file 1 [file 32_398_s1.pdf]

Supplemental material

Anaerobic reduction of nitrate to nitrous oxide is lower in *Bradyrhizobium japonicum* than in *Bradyrhizobium diazoefficiens*

ARTHUR FERNANDES SIQUEIRA,

KIWAMU MINAMISAWA and CRISTINA SÁNCHEZ*

Graduate School of Life Sciences, Tohoku University, 2-2-1 Katahira, Aoba-ku, Sendai 980-8577, Japan

*Corresponding author:

E-mail: cristina.sago@gmail.com; Tel/Fax: +81 022-217-5684

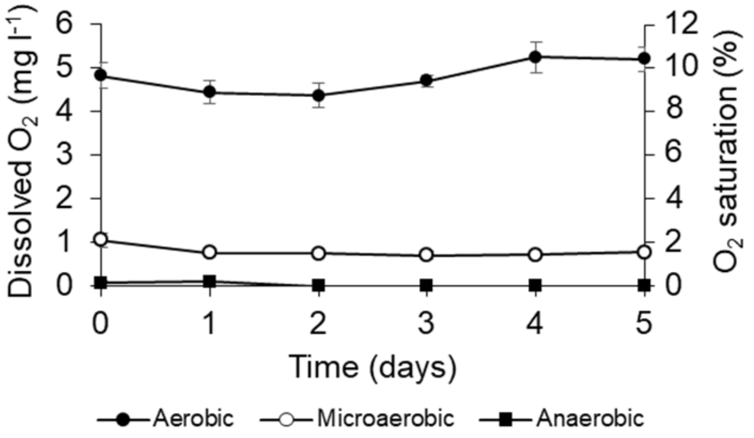

Fig. S1. Levels of dissolved oxygen (mg l^{-1}) and oxygen saturation (%) in aerobic, microaerobic and anaerobic cultures of *B. diazoefficiens* USDA 110^T. Measurements in the microaerobic cultures were performed after replacement of the gas phase (see text). Error bars indicate SE (*n*=3).

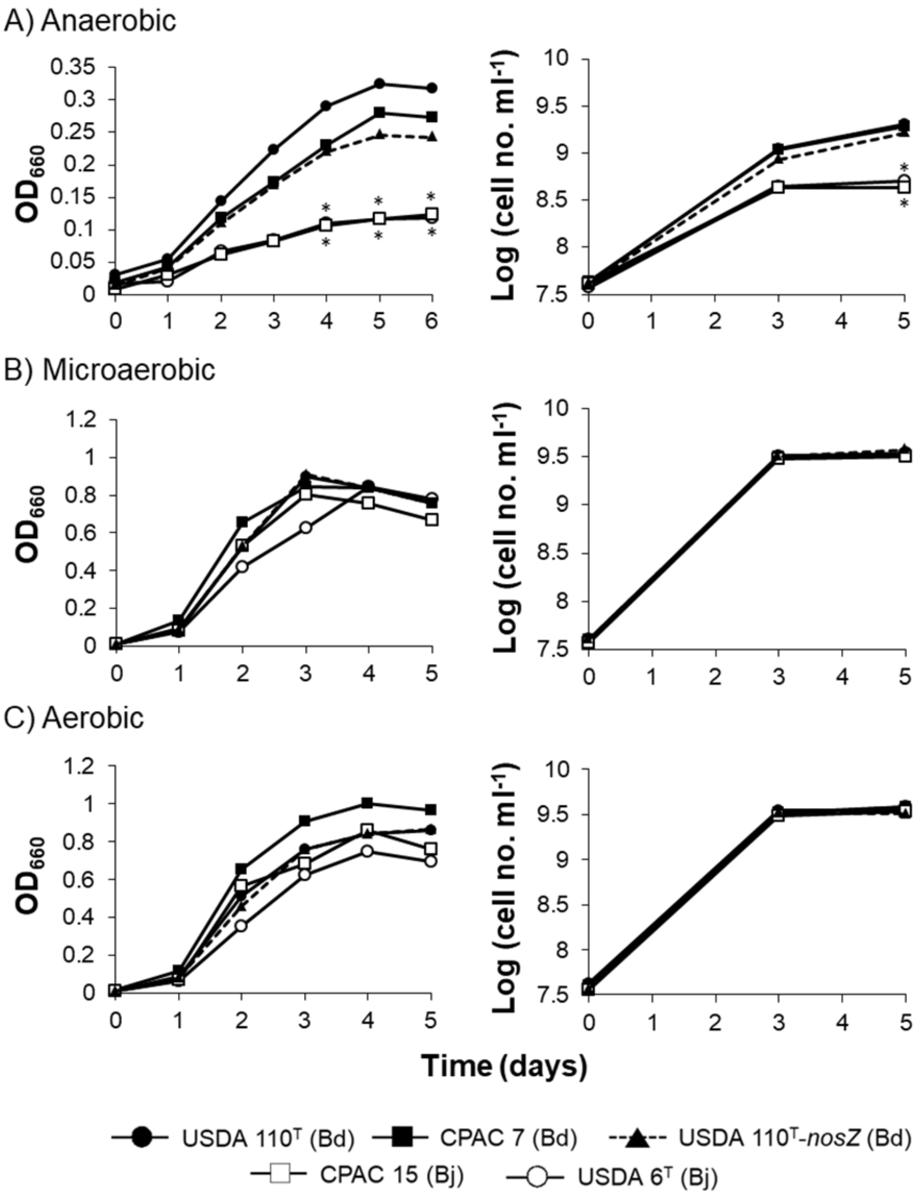

Fig. S2. Anaerobic (A), microaerobic (B) and aerobic (C) growth of *B. japonicum* (USDA 6^T and CPAC 15) and *B. diazoefficiens* (USDA 110^T, CPAC 7, and USDA 110^T-*nosZ* mutant) in HMMN medium. Left panels indicate values of optical density and right panels indicate numbers of cells per ml of culture on a log scale. *Values significantly different from those of *B. diazoefficiens* USDA 110^T (*t*-test, $P<0.05$; $n=3$).

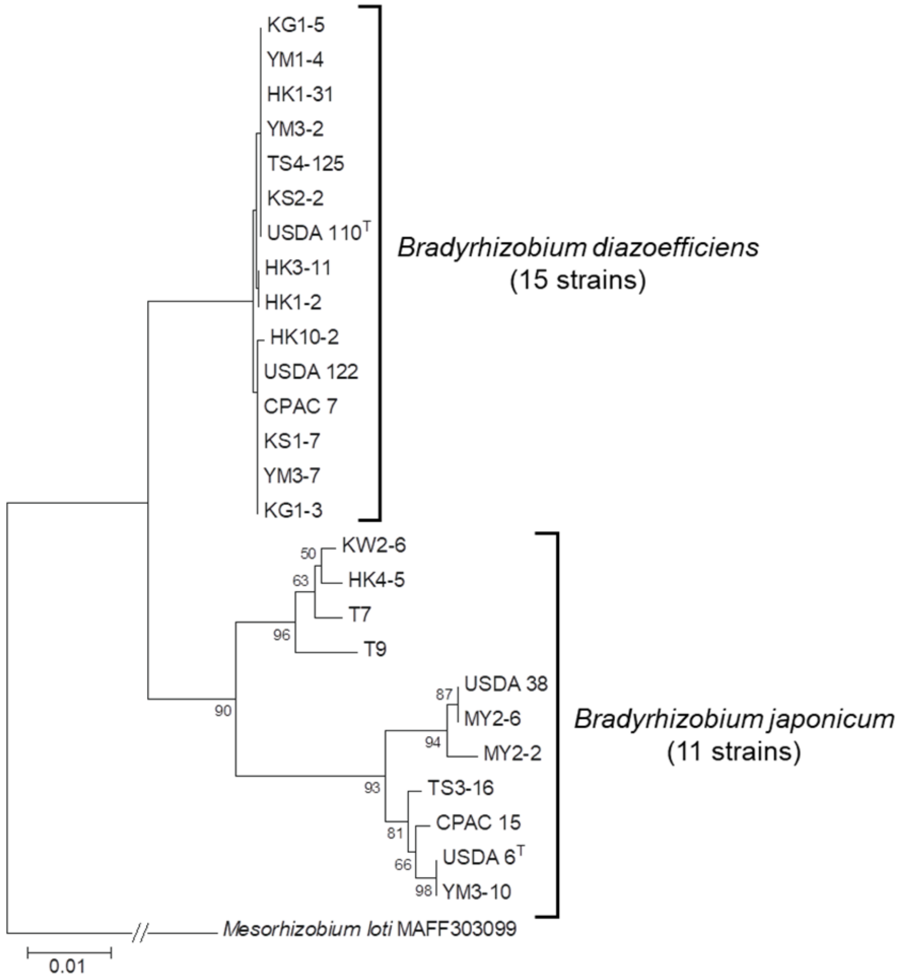

Fig. S3. Neighbor-joining phylogenetic tree based on 16S–23S rRNA internal transcribed sequences showing relationships among the strains used in this study. Bootstrap values >50% (from 1000 replicates) are indicated at the nodes. *Mesorhizobium loti* MAFF303099 was used as an outgroup.

Table S1. Bradyrhizobial strains used in this study.

Strains	Relevant characteristics	Reference
Bradyrhizobium japonicum		
USDA 6^T	Soybean bradyrhizobia, United States Department of Agriculture, National Germplasm Collection, type-strain, *nosZ*-	Kaneko *et al.* (2011)
USDA 38	Soybean bradyrhizobia, United States Department of Agriculture, National Germplasm Collection, *nosZ*-	van Berkum and Fuhrmann (2000)
CPAC 15 (SEMIA 5079)	Soybean bradyrhizobia, Brazilian elite strain inoculant, *nosZ*-	Siqueira *et al.* (2014)
T9	Soybean bradyrhizobia, isolate from Tokachi, Hokkaido, Japan, *nosZ*-	Minamisawa *et al.* (1999)
T7	Soybean bradyrhizobia, isolate from Tokachi, Hokkaido, Japan, *nosZ*-	Minamisawa *et al.* (1999)
KW 2-6	Soybean bradyrhizobia, isolate from Kawatabi, Miyagi, Japan, *nosZ*-	Shiina *et al.* (2014)
MY 2-6	Soybean bradyrhizobia, isolate from Miyazaki, Japan, *nosZ*-	Shiina *et al.* (2014)
YM 3-10	Soybean bradyrhizobia, isolate from Yamagata, Japan, *nosZ*-	Shiina *et al.* (2014)
TU 3-16	Soybean bradyrhizobia, isolate from Tsukuba, Ibaraki, Japan, *nosZ*-	Shiina *et al.* (2014)
MY 2-2	Soybean bradyrhizobia, isolate from Miyazaki, Japan, *nosZ*-	Shiina *et al.* (2014)
HK 4-5	Soybean bradyrhizobia, isolate from Hokkaido, Japan, *nosZ*-	Shiina *et al.* (2014)
Bradyrhizobium diazoefficiens		
USDA 110^T	Soybean bradyrhizobia, United States Department of Agriculture, National Germplasm Collection, type strain, *nosZ*+	Kaneko *et al.* (2002)
USDA 110^T-*nosZ*	Soybean bradyrhizobia USDA 110^T *nosZ*::del/ins Ω cassette	Sameshima-Saito *et al*. (2006)
USDA 122	Soybean bradyrhizobia, United States Department of Agriculture, National Germplasm Collection, *nosZ*+	Sugawara *et al.* (2017)
CPAC 7 (SEMIA 5080)	Soybean bradyrhizobia, Brazilian elite strain inoculant, *nosZ*+	Siqueira *et al.* (2014)
KS 1-7	Soybean bradyrhizobia, isolate from Kashimadai, Miyagi, Japan, *nosZ*+	Shiina *et al.* (2014)
YM 3-7	Soybean bradyrhizobia, isolate from Yamagata, Japan, *nosZ*+	Shiina *et al.* (2014)
HK 10-2	Soybean bradyrhizobia, isolate from Hokkaido, Japan, *nosZ*+	Shiina *et al.* (2014)
KG 1-3	Soybean bradyrhizobia, isolate from Kagoshima, Japan, *nosZ*+	Shiina *et al.* (2014)
KS 2-2	Soybean bradyrhizobia, isolate from Kashimadai, Miyagi, Japan, *nosZ*+	Shiina *et al.* (2014)
TU 4-125	Soybean bradyrhizobia, isolate from Tsukuba, Ibaraki, Japan, *nosZ*+	Shiina *et al.* (2014)
YM 3-2	Soybean bradyrhizobia, isolate from Yamagata, Japan, *nosZ*+	Shiina *et al.* (2014)
HK 1-31	Soybean bradyrhizobia, isolate from Hokkaido, Japan, *nosZ*+	Shiina *et al.* (2014)
KG 1-5	Soybean bradyrhizobia, isolate from Kagoshima, Japan, *nosZ*+	Shiina *et al.* (2014)
YM 1-4	Soybean bradyrhizobia, isolate from Yamagata, Japan, *nosZ*+	Shiina *et al.* (2014)
HK 3-11	Soybean bradyrhizobia, isolate from Hokkaido, Japan, *nosZ*+	Shiina *et al.* (2014)
HK 1-2	Soybean bradyrhizobia, isolate from Hokkaido, Japan, *nosZ*+	Shiina *et al.* (2014)

Table S2. Primers used in this study.

Primer	Sequence (5′→3′)	Source
napA1-f	accaaggtggtctcgttctg	Sánchez *et al.* (2013)
napA1-r	tctttccggtcagaagatgg	Sánchez *et al.* (2013)
nirK_f	cgaggagaagaaggtcgtca	This study
nirK_r	ccttcatgcacgaccatcag	This study
norB_f	taaccagtggctcagcatga	This study
norB_r	tcctgcacgtccatgtaact	This study
sigA-f	gagaaccagatgtcgcttgc	Itakura *et al.* (2013)
sigA-r	tggatgtcctgctcctgaag	Itakura *et al.* (2013)

Table S3. Anaerobic, aerobic, and microaerobic growth of 15 strains of *Bradyrhizobium diazoefficiens* (Bd) and 11 strains of *B. japonicum* (Bj) in HMMN medium for 5 days.

Species	Strain	Days after inoculation*														
		Anaerobic					Aerobic					Microaerobic				
		1	2	3	4	5	1	2	3	4	5	1	2	3	4	5
Bd	USDA 110^T	0.064	0.140	0.235	0.285	0.354	0.068	0.322	0.733	0.799	0.774	0.091	0.282	0.564	0.697	0.699
Bd	USDA 122	0.040	0.099	0.161	0.241	0.268	0.059	0.385	0.912	0.994	0.933	0.083	0.246	0.558	0.868	0.816
Bd	CPAC 7	0.062	0.136	0.240	0.304	0.329	0.071	0.379	0.928	0.923	0.914	0.101	0.356	0.702	0.838	0.798
Bd	KAS 1-7	0.066	0.152	0.263	0.326	0.346	0.091	0.398	0.889	0.868	0.848	0.100	0.351	0.791	0.893	0.856
Bd	YAM 3-7	0.052	0.120	0.210	0.265	0.302	0.059	0.485	1.031	1.016	0.939	0.124	0.380	0.767	0.862	0.829
Bd	HOK 10-2	0.054	0.135	0.226	0.304	0.332	0.057	0.451	1.027	0.985	0.902	0.120	0.341	0.708	0.789	0.734
Bd	KAG 1-3	0.048	0.124	0.208	0.268	0.312	0.073	0.349	0.885	0.934	0.909	0.092	0.347	0.674	0.857	0.817
Bd	KAS 2-2	0.043	0.111	0.184	0.265	0.327	0.055	0.297	0.727	0.785	0.768	0.096	0.281	0.512	0.663	0.665
Bd	TSU 4-125	0.067	0.155	0.238	0.299	0.320	0.083	0.410	0.959	0.907	0.833	0.109	0.345	0.651	0.745	0.740
Bd	YAM 3-2	0.056	0.131	0.203	0.296	0.345	0.062	0.441	1.012	0.886	0.826	0.113	0.342	0.644	0.737	0.741
Bd	HOK 1-31	0.058	0.131	0.221	0.296	0.338	0.055	0.399	0.926	0.885	0.819	0.104	0.307	0.667	0.751	0.733
Bd	KAG 1-5	0.044	0.104	0.156	0.202	0.232	0.053	0.328	0.937	0.981	0.913	0.078	0.248	0.531	0.792	0.747
Bd	YAM 1-4	0.036	0.095	0.172	0.215	0.271	0.068	0.375	0.795	0.754	0.628	0.060	0.210	0.419	0.612	0.601
Bd	HOK 3-11	0.035	0.124	0.213	0.295	0.340	0.058	0.380	0.936	0.923	0.851	0.070	0.297	0.560	0.745	0.713
Bd	HOK 1-2	0.049	0.134	0.222	0.295	0.337	0.030	0.324	0.845	0.893	0.854	0.081	0.270	0.551	0.753	0.720
Bj	USDA 6^T	0.017	0.068	0.113	0.132	0.147	0.041	0.223	0.450	0.675	0.711	0.046	0.204	0.385	0.615	0.669
Bj	USDA 38	0.025	0.082	0.118	0.125	0.123	0.022	0.247	0.728	0.967	0.866	0.042	0.202	0.452	0.760	0.720
Bj	T9	0.010	0.020	0.062	0.143	0.177	0.014	0.130	0.463	0.831	0.808	0.025	0.088	0.197	0.304	0.530
Bj	T7	0.036	0.084	0.130	0.176	0.194	0.062	0.439	0.984	1.074	1.046	0.075	0.341	0.733	0.881	0.870
Bj	CPAC 15	0.050	0.113	0.177	0.211	0.221	0.128	0.556	0.979	0.910	0.885	0.094	0.385	0.731	0.744	0.691
Bj	KAW 2-6	0.031	0.071	0.102	0.119	0.114	0.068	0.414	0.809	0.868	0.828	0.078	0.276	0.516	0.713	0.687
Bj	MIY 2-6	0.026	0.119	0.181	0.196	0.193	0.043	0.272	0.678	0.817	0.797	0.040	0.249	0.558	0.849	0.821
Bj	YAM 3-10	0.021	0.057	0.088	0.131	0.100	0.041	0.382	0.822	0.932	0.861	0.074	0.289	0.623	0.837	0.785
Bj	TSU 3-16	0.028	0.085	0.126	0.144	0.151	0.146	0.412	0.705	0.850	0.811	0.074	0.306	0.502	0.773	0.716
Bj	MIY 2-2	0.021	0.077	0.123	0.151	0.166	0.035	0.323	0.825	0.892	0.788	0.057	0.263	0.527	0.741	0.661
Bj	HOK 4-5	0.018	0.081	0.113	0.154	0.185	0.022	0.178	0.512	0.826	0.873	0.042	0.126	0.257	0.501	0.546

*Values are means of three biological replicates.

References

Itakura, M., Uchida, Y., H. Akiyama, *et al.* 2013. Mitigation of nitrous oxide emissions from soils by *Bradyrhizobium japonicum* inoculation. Nat. Clim. Change. 3:208-212.

Kaneko, T., Y. Nakamura, S. Sato, *et al.* 2002. Complete genomic sequence of nitrogen-fixing symbiotic bacterium *Bradyrhizobium japonicum* USDA110. DNA Res. 9:189-197.

Kaneko, T., H. Maita, H. Hirakawa, N. Uchiike, K. Minamisawa, A. Watanabe, and S. Sato. 2011. Complete genome sequence of the soybean symbiont *Bradyrhizobium japonicum* strain USDA6^T. Genes. 2:763-787.

Minamisawa, K., Nakatsuka, Y., and T. Isawa. 1999. Diversity and field site variation of indigenous populations of soybean bradyrhizobia in Japan by fingerprints with repeated sequences RSα and RSβ. FEMS Microbiol. Ecol. 29:171-178.

Sameshima-Saito, R., K. Chiba, J. Hirayama, M. Itakura, H. Mitsui, S. Eda, and K. Minamisawa. 2006. Symbiotic *Bradyrhizobium japonicum* reduces N_2O surrounding the soybean root system via nitrous oxide reductase. Appl. Environ. Microbiol. 72:2526-2532.

Sánchez, C., M. Itakura, H. Mitsui, and K. Minamisawa K. 2013. Linked expressions of *nap* and *nos* genes in a *Bradyrhizobium japonicum* mutant with increased N_2O reductase activity. Appl. Environ. Microbiol. 79:4178-4180.

Shiina, Y., M. Itakura, H. Choi, Y. Seaki, M. Hayatsu, and K. Minamisawa. 2014. Relationship between soil type and N_2O reductase genotype (*nosZ*) of indigenous soybean bradyrhizobia: *nosZ*-minus populations are dominant in Andosols. Microbes Environ. 29:420-426.

Siqueira A.F., E. Ormeño-Orrillo, R.C. Souza, *et al.* 2014. Comparative genomics of *Bradyrhizobium japonicum* CPAC 15 and *Bradyrhizobium diazoefficiens* CPAC 7: elite model strains for understanding symbiotic performance with soybean. BMC Genomics. 15:420.

Sugawara, M., T. Tsukui, T. Kaneko, Y. Ohtsubo, S. Sato, Y. Nagata, M. Tsuda, H. Mitsui, and K. Minamisawa. 2017. Complete genome sequence of *Bradyrhizobium diazoefficiens* USDA 122, a nitrogen-fixing soybean symbiont. Genome Announc. 5: e01743-16.

van Berkum, P. and Fuhrmann, J.J. 2000. Evolutionary relationships among the soybean bradyrhizobia reconstructed from 16S rRNA gene and internally transcribed spacer region sequence divergence. Int. J. Syst. Evol. Microbiol. 50:2165-2172.
